# Design guidelines for assessing students’ interprofessional competencies in healthcare education: a consensus study

**DOI:** 10.1007/s40037-022-00728-6

**Published:** 2022-10-12

**Authors:** Hester Wilhelmina Henrica Smeets, Dominique M. A. Sluijsmans, Albine Moser, Jeroen J. G. van Merriënboer

**Affiliations:** 1 Research Centre for Autonomy and Participation, Zuyd University of Applied Sciences, Heerlen, The Netherlands; 2 School of Health Professions Education, Maastricht University, Maastricht, The Netherlands; 3 Research Centre Urban Talent, Rotterdam University of Applied Sciences, Rotterdam, The Netherlands; 4 Department of Family Medicine, Maastricht University, Maastricht, The Netherlands

**Keywords:** Interprofessional education, Interprofessional assessment, Competency-based assessment, Healthcare education

## Abstract

**Introduction:**

Healthcare systems require healthcare professionals and students educated in an interprofessional (IP) context. Well-designed assessments are needed to evaluate whether students have developed IP competencies, but we currently lack evidence-informed guidelines to create them. This study aims to provide guidelines for the assessment of IP competencies in healthcare education.

**Methods:**

A qualitative consensus study was conducted to establish guidelines for the design of IP assessments using the nominal group technique. First, five expert groups (IP experts, patients, educational scientists, teachers, and students) were asked to discuss design guidelines for IP assessment and reach intra-group consensus. Second, one heterogeneous inter-group meeting was organized to reach a consensus among the expert groups on IP assessment guidelines.

**Results:**

This study yielded a comprehensive set of 26 guidelines to help design performance assessments for IP education: ten guidelines for both the IP assessment tasks and the IP assessors and six guidelines for the IP assessment procedures.

**Discussion:**

The results showed that IP assessment is complex and, compared to mono-professional assessment, high-quality IP assessments require additional elements such as multiple IP products and processes to be assessed, an IP pool of assessors, and assessment procedures in which standards are included for the IP collaboration process as well as individual contributions. The guidelines are based on expert knowledge and experience, but an important next step is to test these design guidelines in educational practice.

**Supplementary Information:**

The online version of this article (10.1007/s40037-022-00728-6) contains supplementary material, which is available to authorized users.

## Introduction

Health professionals should collaborate with professionals from other health domains [1]. This necessitates embedding interprofessional (IP) education within the curricula of academic healthcare institutions. IP education (IPE) focuses on teaching students how to learn from, with, and about other professionals to improve collaboration and, eventually, the quality of care [2]. Well-designed IP assessments are needed to make valid inferences about students’ IP collaborative competencies (e.g., IP communication, role clarification, collaborative leadership and interprofessional conflict resolution) [3, 4].

However, measuring IP competencies is complex [5] because competencies require a holistic judgment that incorporates the knowledge, skills, and attitudes demonstrated in a clinical context and influenced by this context and the learning process [6, 7]. Well-designed performance assessments should focus on students’ abilities to use combinations of skills, knowledge, and attitudes [8]. To ensure that the assessment is fit for purpose, an assessment approach is required that concentrates on the design of performance assessments.

Assessment is currently the weakest link in undergraduate IPE [5, 9], and a systematic design approach in assessment practice is lacking [10]. Despite the available knowledge about the separate constructs of IP assessments (e.g., assessment tasks, rating tools) and the design of performance assessments, there is little understanding of how to design coherent IP assessments. There seems to be a strong focus on assessment instruments, such as questionnaires and rubrics. This can lead to a narrow approach to the design of IP assessments and forgetting about other important assessment aspects, such as the characteristics of the assessors or rules to decide on a student’s performance [5, 9, 10]. Designing performance assessment goes beyond the assessment instrument, but focuses on a procedure to make inferences in which the constellation of tasks, assessors, and decision rules also contribute to the quality of the assessment [11]. Therefore, this study aims to formulate design guidelines for the assessment of students’ IP competencies.

There are significant challenges to the design of IP assessments. First, there is the diversity of the assessment tasks used. An IP assessment task is an educational task in which students from two or more educational programs show their IP competence, such as simulation-based tasks and case-based discussions [10]. The IP tasks vary in quality, focus, and theoretical underpinning [9, 12, 13] and as a result, there is little consensus regarding an “end-level” of IPE, stating when students are collaboration-ready. Second, an IP assessor is responsible for assessing, examining, and/or grading students’ IP performance. Yet, we lack insight into the desired quantity and nature of assessors—for example, whether these are teachers, professionals, or peers—and their specific characteristics, such as their training and required level of assessment-specific expertise [5, 14]. The third challenge regards IP assessment procedures (i.e., appropriate assessment formats to rate students’ performance and rules for evaluating student progress) [15]. The most important aspect of performance assessment is defining the criteria in observable terms [11]. It is often unclear what the content of IP collaboration is to be assessed. Few tools are available for undergraduate IP assessment, and existing instruments often measure merely one aspect of competence, such as “attitude” [5, 16, 17]. A specific difficulty regarding the IP assessment procedure is the feasibility of assessment. Often hundreds of learners are to be assessed and realistic methods that address the diversity of tasks, the assessors, and assessment formats must be considered [12].

What distinguishes IP assessments from mono-professional assessment is the question who should be assessed. In the IP assessment literature, the current discussion revolves around whether to assess individual students, the IP team, or both. We see that assessment of individual competencies is currently dominant in healthcare education [12]. Assessment of an IP team can be challenging. In every IP team, students with different domain-specific competencies participate and they invest different amounts of time and effort into the collaboration [18]. One team member’s abilities may influence the performance of an entire team. It is unclear how individual competence can be assessed through team performance and what implications the performance of an individual has on the performance of the whole team.

A recent scoping review of current IP assessment literature [10], demonstrated that evidence-informed guidelines are missing for the design of IP assessments, including guidelines for assessment tasks, assessors, and assessment procedures. Thus, we aim to answer the following research question in this study:

What are the guidelines for the design of (1) the assessment tasks, (2) the pool of assessors, and (3) the assessment procedures for assessing students’ IP competencies?

## Methods

### Design

A qualitative consensus study was conducted using the nominal group technique (NGT) [19]. We chose the NGT because we wanted participants to generate ideas together for IP assessment guidelines and prioritize them using face-to-face discussion. This study consisted of two phases: an intra-group consensus phase, in which consensus was sought *within* five expert groups; and an inter-group consensus phase, in which consensus was sought *among* the five expert groups. Ethical approval was granted by the research ethics committee from the Faculty of Health, Medicine, and Lifestyle, Maastricht University (approval no. FHML-REC/2019/078).

### Intra-group consensus study

#### Participants

We enrolled five expert groups to represent distinct perspectives: international experts on IPE (*n* = 6), educational scientists (*n* = 7), patients (*n* = 5), students (*n* = 9), and teachers (*n* = 7) (see Electronic Supplementary Material 1 [ESM 1] for detailed participant information). Most experts were recruited from the Netherlands, although we included experts from Brazil, the United Kingdom, Sweden, Finland, and Belgium to provide international perspectives.

We started with a purposive sampling strategy [20] aiming to include participants who had knowledge and expertise regarding IP collaboration and IP assessment and participants who had been involved in IPE. Participants had to have a positive attitude towards IPE and meet our inclusion criteria (Tab. 1). Since we preferred face-to-face interaction between participants, we subsequently used convenience sampling. Convenience sampling was used to reach participants of our target population who had met practical criteria, such as easy accessibility, availability at a given time, and willingness to participate [20]. For example, IP experts were sampled at an international IP conference. The total participant group was fit for purpose and designed to represent a wide range of viewpoints and expertise regarding IP assessments.

**Table 1:** Inclusion criteria

IPE expert group	Inclusion criteria
IPE experts	– A minimum of two years of experience with IPE – Involvement in research regarding IPE
Educational scientists	– A minimum of one year of experience in instructional design or performance-based assessment – Experience in educational research
Patients	– A minimum of two years of involvement in IPE – Involvement in higher education
Students	– A minimum of two years of involvement in IPE, with a minimum of participation in two IP educational activities (e.g., an IP educational module or an IP internship)
Lecturers	– A minimum of two years of involvement in IPE and IP assessment – Experience with working in an IP practice

#### Materials

We developed an introductory video to provide participants with background information about the study, explain all relevant definitions, and introduce a preparatory assignment. In this assignment, participants were asked to think about IP assessment in relation to IP tasks, assessors, and procedures in advance, using their prior knowledge or experience in the field of IPE or assessment. Participants were asked to write down any ideas regarding the research question and bring them to the session.

We developed an interview guide for each consensus session, including questions to be asked and practical information (See ESM 2 for the interview guide). We collected participants’ sociodemographic details via the questionnaire, including their IPE experiences, to provide a comprehensive overview of who participated and check for any confounding effects.

#### Procedure

Data were collected between September 2019 and April 2020. We organized five homogenous NGT sessions with the expert groups. Each NGT session lasted 90–120 min and was moderated and observed by research team members.

To begin the NGT session, the moderator asked participants to present their guideline ideas from the preparatory task on IP assessment consecutively in a round-robin format. The observer wrote all guideline ideas on post-it notes and subsequently organized them on three separate posters based on IP tasks, assessors, and procedures. We classified these as draft guidelines. Participants briefly discussed the draft guidelines and outlined any disagreements. Subsequently, each participant received five stickers for IP tasks, assessors, and procedures each, to place on the draft guidelines they found most relevant. All NGT sessions were audio-taped and transcribed verbatim.

#### Data analysis

We analyzed the data using eight steps for deductive and inductive content analysis [21]. As preparation, two research team members (HS & AM) read the transcripts multiple times for data immersion. Next, we selected transcript fragments that were meaningful for answering our research question. We then started with a deductive approach for which an analysis matrix was developed based on the three main challenges in IP assessment tasks, assessors, and procedures [10, 14]. All data were reviewed for content and coded for correspondence with the research question. Subsequently, we used inductive coding, in which we wrote notes and headings in the text, freely generating new codes, categories, and subcategories. The next step consisted of grouping the lists of codes, categories, and subcategories under higher-order headings. Next, we formulated a general description of the lists of categories, using content-characteristic words. We finally compared the categories of the five transcripts to identify similarities and differences in guidelines per expert group.

The research team compared the draft guidelines for IP assessment in each of the five homogenous expert sessions and created two documents. The first document included draft guidelines for which there was consensus among two or more expert groups and was regarded as consented and presented for the final member check. The second document presented draft guidelines for which there was no consensus or consensus in only one expert group and was used as input for the *inter-group* consensus session, to discuss which guidelines should be added to the final list. Atlas.ti (https://atlasti.com/, version 8.4.24) was used to organize the data.

### Inter-group consensus

The second phase consisted of one inter-group consensus session, in which representatives from all five expert groups discussed the guidelines for which no consensus was reached or consensus in only one expert group, and reached a final consensus on the IP assessment guidelines.

#### Participants

Ten participants from the previous sessions attended the inter-group consensus session: one IPE expert with a background in physiotherapy (from Belgium, participating digitally); three patients; one educational scientist, with a background in medicine and with expertise in competency-based assessments; three occupational therapy, speech therapy, and physiotherapy teachers; and two nursing students.

#### Materials

Materials were similar to the intra-group consensus session with the addition of a laptop to enable remote participants’ online participation. Participants were provided the draft guidelines for which there was either consensus (handout 1) or no/limited consensus (handout 2) to provide insight into these guidelines.

#### Procedure

The moderator initiated a round-robin exercise where participants consecutively responded to the first set of draft guidelines for which no consensus was reached in the first round. In this session, we held a discussion to reach a consensus regarding the intra-group differences that arose in the previous phase. The moderator ensured that all participant groups were involved in the discussion. The moderator asked the participants to prioritize the draft guidelines for which no consensus had been reached, using three stickers per research question. After the final consensus session, we organized a single member check session with three (vice-)chairs of international IP organizations who participated in the study. They reflected on the clarity and usability of the final guidelines.

#### Data analysis

We analyzed the inter-group consensus data by checking whether the codes from the inter-group consensus session fit into formulated subcategories and categories related to the research question. We either created a new subcategory, which was the case for two subcategories, or adapted the description of the subcategories. The analysis resulted in nine subcategories for each research question regarding the assessment tasks (*n* = 4), the assessors (*n* = 3), and the procedures (*n* = 2) (ESM 3).

### Trustworthiness

Trustworthiness was pursued by applying strategies regarding credibility and transferability [22]. Credibility was established by source, investigator and analyst, and data triangulation. Source triangulation was achieved by working with five different expert groups. Investigator and analyst triangulation was achieved by working with multiple researchers in the NGT sessions, then analyzing sections of the data with at least three researchers. Data triangulation was achieved by working with raw data (audio files), multiple transcripts, reflective notes, and summaries of the NGTs. In addition, we member-checked whether the interpretations of data were correct by sending the participants a summary after each session.

## Results

### Intra-group consensus

#### IP assessment tasks

##### IP competency framework

Participants believed it was critical to formulate learning goals at the beginning of the IP module as input for the IP assessment and to assign all participating students the same goals and assessment tasks. They emphasized that IP assessment tasks should cover a broad array of competencies, such as IP communication skills and the ability to think beyond their own profession when working on an IP case.

##### Conditions of the IP task

Participants emphasized that “*It must be clear to students whether this is an assessment for learning or whether it is a high-stake evaluation with consequences”* [IPE expert A]*. *They prioritized that students should be aware of the learning goals and assessment criteria, so that students are informed about what should be done to pass the IP assessment. They also found it important that IP assessment tasks be performed when taking their professional roles, because: *“No one can assess if I did a good job because we are sitting together only with nurses, who cannot assess if I made good occupational diagnoses” *[Student I]*.*

##### Characteristics of the IP task

Participants found it essential to include the word “IP team” in the IP assessment task to ensure transparency about everyone’s responsibilities and roles. They also stressed that students have to work together on a case or product: *“A play is maybe a good metaphor to define IP tasks. I think the crux is that we are all needed at the same time; we have to work together for the patient” *[Educational scientist B]*. *Next to this, the assessment tasks should be authentic and resemble IP workplace practices.

##### IP performance

Participants felt it was essential that students be assessed on both the product and process of IP collaboration. As outcomes of the IP assessment task, there should be a joint assessment in which both the students’ product and the IP process leading to it are evaluated.

#### IP assessors

##### Assessor characteristics

Participants prioritized that students should receive feedback from multiple assessors. These assessors should then be able to transcend their professional perspectives or context, and should be aware of the students’ different professional backgrounds in the IP assessment task.

Training in the assessment of IP competencies and (life) experience are important characteristics, because: *“Well, I connect this life experience to someone … who has seen a lot, who is hardened by what he sees. So, assessors have multiple years of experience, which means that they are better able to assess the situation” *[Patient D].

Participants indicated that patients, or simulation patients, should have a role on the assessor team when patients could think in a “disease-transcending” manner. That entails that patients acknowledge their illness, can look beyond possible limitations, and are able to speak to students about their condition when participating in education. Participants believed that IP assessors need calibration sessions to acquaint and train all assessors in using the assessment.

##### Knowledge, skills, and attitudes

Participants concurred that the IP assessors should have IP collaboration experience, either working as a professional or at an educational institution. They saw IP assessors as role models for the students, that they “*walk the talk*” [IPE expert C], and participants thought that IP assessors should have a positive attitude regarding IPE.

##### Conditions of the pool of IP assessors

Participants found it important to work with at least two IP assessors. Further, they thought that assessors should conduct assessments independently, compare and discuss IP assessment results, and come to a joint decision using the same criteria to assess students.

#### IP assessment procedures

##### IP assessment instrument

When an assessment instrument is used, participants believed that *“high-quality assessment instruments and corresponding performance criteria are needed”* [Teacher G]. For the criteria on which students are assessed, they found it critical that these matched the criteria used in IP workplace practice, and that these criteria should be concise and straightforward.

##### Conditions of the IP assessment procedure

Participants indicated that an IP competency framework is fundamental for IP assessment procedures. Participants furthermore prioritized that the assessment procedure should consist of a mix of multiple assessments of acquired IP competencies that are combined to make progress decisions.

### Inter-group consensus

The outcome after the inter-group consensus session is presented in Tab. 2. The participants reached a consensus on several draft guidelines without discussion (NC1, NC4–6, NC10–13, NC15, NC16).

**Table 2:** Draft guidelines with no intra-group consensus

Draft guideline	Outcome
**Assessment Task**	
*NC1* Use an IP competency framework that serves as the basis of the IP assessment, based on the final qualifications, suiting each profession	C
*NC2* The assessment task entails both a summative assessment and formative evaluations in which the students receive feedback on their interprofessional competencies	CD
*NC3* The assessment task is adapted to the differences among the students involved	NCD
*NC4* The student performs the assessment task in their professional role	C
*NC5* The IP assessment task is the same for all professions	C
NC6 In the design of the assessment task, the differences in professional language are considered	C
*NC7* A starting point of the assessment task is that there should be shared responsibility among students	NCD
**Assessor**	
*NC8* The team of assessors is competent in educational assessment	NCD
*NC9* A patient is part of the pool of assessors	CD
*NC10* The IP assessors are trained in the use of the assessment instrument	C
*NC11* The IP assessors are experienced in the field of their professional practice	C
*NC12* The team of assessors is aware of the learning goals, the assessment task, the assessment instrument, and the criteria for student assessments	C
*NC13* Each assessor uses the same procedure to assess the IP competencies of the students	C
*NC14* The pool of IP assessors has a moderation process and calibration sessions as part of the assessment procedure	CD
*NC15* Educational programs ensure that assessors from different professions can assess students	C
**Assessment procedure**	
*NC16* The assessment instrument is based on the competencies on which the students are assessed	C
*NC17* The assessment program increases in complexity regarding IP assessment from the first to last year of the educational program	NCD
*NC18* Educational credits should be awarded to the students who pass the IPE	CD
*NC19* In the assessment procedure, the collaboration process and the individual contributions of the students are included	CD

*NC* no consensus, *C* consensus, *CD* consensus after discussion, *NCD* no consensus after discussion

#### IP assessment tasks

A discussion among participants focused on the terminology of summative and formative assessments (NC2) and whether feedback should be an integral element of IP assessment. It appeared that at different educational institutions, different terms were used to define the function of the assessment: *“You can have endless discussions about this aspect of language, but I think the basis is that you want to give feedback to the student ‘during the ride’ on process and product, and, at the end, you assess a process and a product” *[Educational scientist A].

The draft guideline stating that shared responsibility is needed in IP assessment (NC7) led to a discussion about whether shared responsibility should be the aim of the assessment task. According to participants, the starting point for the tasks should be authentic IP situations, in which a shared responsibility among students is required to complete their assignment.

#### IP assessors

Participants discussed whether the team of assessors in IPE should be competent in educational assessment (NC8). They concurred assessors in IPE must be trained and instructed at the start of the IPE course, which they found more important than having much assessment expertise.

Participants discussed whether patients should be part of the assessor team (NC9). On the one hand, most participants thought patients should be part of the assessor team because: *“In the educational module, I never see a report that the students make. I show up three times, I show my face, I tell my story, and what happens after that? I hope to see that in practice, but I think it is a pity that I do not have to say something about their IP product” *[Patient B]. On the other hand, some patients expressed doubt about this. In the consensus session, participants added that patients should be involved, but only if they want to play a role in the assessment (e.g., an advisor assessing a student’s acquired IP competencies).

Participants’ ideas differed about whether to calibrate between assessors before student evaluations or include a moderation process afterward (NC14). Participants believed it vital to hold calibration sessions at the start of the IPE among all assessors to ensure that the assessment procedures are clear to everyone.

#### IP assessment procedures

Participants discussed whether the IP assessment should be imperative for all participating professions and whether the IPE should be embedded in the curricula instead of rewarded with additional credits (NC18), however they explained that: *“I would dare to formulate a guideline stating that it is highly recommended that educational credits are awarded to students. Then, it has an obligatory character, in which participating programs are viewed equally” *[IP expert F].

The final discussion revolved around whether the IP team or the individual should be assessed (NC19). Participants agreed that it does not matter whether you assess the team or the individual, as long as you are transparent to the students about the procedures. However, in the member check session, the three participants believed it to be of high importance that procedures are considered in the IP assessment for both the collaboration process of the team as well as individual contributions. Therefore, this was added to the final set of guidelines. Tab. 3 presents the guidelines for the design of IP assessments.

**Table 3:** Guidelines for the design of a program to assess IP competencies

Guidelines for the design of assessments for IP education *With these guidelines, we aim to address the design team in charge of designing the IP assessment program, which can consist of lecturers, managers, students, policy makers, patient(representative)s, and other stakeholders*		
IP Assessment Task^a^	IP Assessors^b^	IP Assessment Procedure^c^
The IP assessment task should be based on a description of the required IP competencies	The team of assessors should consist of multiple relevant actors, such as peers, patients (if willing and able to participate), professionals, and lecturers	In the assessment procedure, the standards should be clear, concise, and transparent
The IP assessment task should be based on the professional qualifications as defined in each professional profile	During the course, the team of assessors should provide feedback to students about their progress regarding performance outcomes	In the assessment procedure, the standards are aligned with the performance outcomes of the IP course
In the IP assessment task, there should be clear and transparent communication about the way students are assessed (which competencies, why, how, function)	The roles (tasks and responsibilities) of each assessor on the assessment team should be clearly defined	The assessment procedure should include rules on how feedback is included to reach a decision about the acquisition of IP competencies
In the IP assessment task, both the task and the underlying performance outcomes should be the same for all participating students regardless of professional background	The team of assessors should be informed about performance outcomes, the assessment task, the assessment instrument, and the standards on which students are assessed	In the assessment procedure, standards are included on the quality of the IP collaboration process and the individual contribution of students to it
The IP assessment task should describe an authentic professional (patient) case in which multiple professions must collaborate to solve the task	The team of assessors should include at least one assessor with: practical experience as a healthcare professional, practical experience in interprofessional collaboration, interprofessional competence	Students should be rewarded with credits when passing the IP assessment task
The IP assessment task should be carried out by the students based upon their professional background	The team of assessors should be trained in the assessment procedure used	The IP assessment is embedded in students’ educational programs
The IP assessment task should lead to both products and processes as performance outcomes	To have a shared understanding and interpretation of the assessment standards, the team of assessors should hold calibration sessions before the assessment	
The IP assessment task should require student reflection on the quality of the IP collaboration process	The team of assessors should understand which assessment procedure is used to decide on IP competencies and should adhere to this model	
The IP assessment task should include multiple opportunities for feedback on students’ development	The team of assessors should be facilitated in time and resources to conduct the assessment	
In the IP assessment task, language should be used that can be understood by all participating students	The team of assessors should be facilitated by the educational programs to assess students from different professions	

^a^ An IP assessment task is an educational task in which students from two or more educational programs show their IP competence. This IP assessment task leads to IP performance that can be assessed.

^b^ An IP assessor is responsible for assessing/examining/grading the IP performance of the students.

^c^ An IP assessment procedure describes the performance criteria and decision rules based on which the assessors can judge the IP competencies of students.

## Discussion

This study developed 26 guidelines for the design of coherent interprofessional assessments in healthcare education that address IP assessment tasks, assessors, and assessment procedures. This study contributes to the body of knowledge by proposing guidelines for comprehensively designing IP assessments, in contrast to other publications focusing mostly on one aspect of an IP assessment.

Concerning IP tasks, the proposed guidelines align with previous studies, e.g., the authenticity of the assessment tasks [23–26], the importance of transparency in describing the purpose of the assessment and the criteria applied to students [8, 27, 28], and the identification of IP competencies on which the assessment task is based [12, 14]. Regarding assessors, the guidelines generated state that the assessors should be aware of the assessment content and process and trained in the use of the assessment procedure, which is confirmed by several other publications [5, 9, 12]. The guideline regarding the facilitation of assessors to evaluate a student from a different profession is relatively new in IP assessment literature since it is often the case that educational institutions require students to be assessed by teachers within their profession [9, 14].

Regarding the assessment procedures, many assessment instruments are available in current literature [29], however, the instruments used are not always based on the IP competencies underlying the IP assessment and IP performance criteria [10]. Based on our findings, we recommend searching for or developing assessment instruments aligned with the purpose of the assessment and the IP performance criteria which the assessment focuses on.

There seems to be disagreement in the IP literature whether IP assessments should focus on the assessment of the IP team, on the individual’s contribution to the team, or—as advised by the study participants—both [14, 28]. Assessments of individual competencies are still dominant in healthcare, especially for undergraduate student assessments for certification [10]. However, some issues occur when assessing only the team of students, such as free-riding behavior, and when assessing only the individual, such as competition among students [18]. These behaviors do not lead to the objective of the assessment: IP collaborative learning. This study shows that it is necessary to transform assessment from an individual-based approach to an IP approach in health professions education [9, 12]. It seems crucial to develop new techniques focusing on the IP performance of individuals and the whole IP team [5, 14]. More research is needed on the assessment of team performance, especially regarding the implications of individual performance on the performance of the team, and how to draw inferences about individual competence, based on the performance of an IP team.

The guidelines formulated in this study imply integrated IP assessment, using multiple assessment tasks, assessors, and tools. IP competencies are complex and consist of several sub-competencies, which can hardly be achieved using one IP assessment method. The literature also suggests improving assessment of such complex competencies by creating an integrated set of assessments instead of relying on one single assessment for an overall decision [11, 28, 30–32]. However, it is challenging to use multiple IP assessment tasks and tools in a single IPE course since IPE is often merely a small part of healthcare curricula [14]. Ideally, IP assessment development is balanced across a curriculum, with different IP assessment tasks focusing on distinct but overlapping clusters of IP competencies [5]. To advance IP assessment, more research is needed to determine which IP competencies should be at the core of the undergraduate healthcare curricula, at which complexity level, and how they should be assessed to determine what “ready for collaborative practice” entails. When focusing on multiple IP competencies, more research is needed to ascertain how an integrated set of assessment tasks, assessors, and tools can be designed while taking into account the costs and the proportion of IPE in the curriculum as a whole.

Our study has several strengths and limitations. One strength is that we started with homogenous sessions to ensure that multiple, relevant perspectives from the same group were considered. In the heterogeneous consensus session, representatives from all expert groups engaged in an IP discussion. The combination of both session types enabled consideration of each idea, leading to a final consensus on the most important guidelines. Another strength is the expertise and variety of the expert groups. We considered all IP expert groups equally important. Thus, we gathered rich data about IP assessment from many viewpoints. Our purposive sampling strategy focused on knowledge and expertise regarding IP collaboration and IPE. A limitation of this study was that we did not sample according to professional backgrounds, which led to an exclusion of students and teachers with a medical background. Ideally, there would be uniform representation amongst the groups reflecting the spectrum of IPE disciplines. Nonetheless, in this study, the medical background has been represented. Because of the diversity of knowledge and expertise in our participant groups, the guidelines can be transferred to and used in other settings, such as in medical education. Another limitation is that we started with deductive analysis based on the three assessment elements, namely, tasks, assessors, and procedures. We might have overlooked information relating to other assessment elements (e.g., the reason for the assessment). We recommend using the guidelines from this study in educational practice in combination with theory on designing performance assessments [11].

## Supplementary Information

ESM 1 More detailed information about the participants from the five expert groups who participated in this study

ESM 2 Qualitative interview guide used in this study

ESM 3 Figure that gives an overview of the categories per assessment element tasks, assessors, and procedures, that followed the analysis of the data
