# Supplementary material for: Design guidelines for assessing students’ interprofessional competencies in healthcare education: a consensus study
Source: Perspect Med Educ. 2022 Oct 12;11(6):316–24. doi: 10.1007/s40037-022-00728-6 (PMC9743853; doi:10.1007/s40037-022-00728-6)
Supplement: Supplementary file 1 — ESM 1 More detailed information about the participants from the five expert groups who participated in this study [file 40037_2022_728_MOESM1_ESM.docx]

**Electronic Supplementary Material (ESM) 1**

Characteristics of expert groups

Characteristics of expert group: Patients

| **Patient** | **Age range** | **Gender** | **Years of experience as a patient** | **Experience in education** |
| --- | --- | --- | --- | --- |
| A | 30 – 40 years | F | 12 years | Lecturers at higher education institutes about condition & sharing story with students in higher healthcare education |
| B | 50 - 60 years | M | 23 years | Lecturers at higher education institutes about condition & sharing story with students in higher healthcare education |
| C | 50 - 60 years | F | 17 years | Involved in interprofessional education research projects |
| D | 60+ years | M | 9.5 years | Sharing story with students in higher healthcare education |
| E | - | F | - | Sharing story with students in higher healthcare education |

Characteristics of expert group: IP experts

| **IP expert** | **Age (range)** | **Gender** | **Years of experience in IP education** | **Years of experience in education** | **Professional background** |
| --- | --- | --- | --- | --- | --- |
| A | 50-60 years | F | 6 years | 15 | Medicine |
| B | 40-50 years | F | 3 years | 16 years | Physiotherapy |
| C | 60+ years | M | 23 years | 35 years | Nursing |
| D | 60+ years | F | 15 years | 25 years | Public health |
| E | 60+ years | F | - | - | Clinical chemistry |
| F | 40-50 years | F | 7 years | 10 years | Physiotherapy |

Characteristics of expert group: Teachers

| **Teacher** | **Age (range)** | **Gender** | **Years of experience in education** | **Professional background** |
| --- | --- | --- | --- | --- |
| A | 40 - 50 | F | 22 years | Nursing & health sciences |
| B | 60+ | F | 24 years | Physiotherapy |
| C | 20 – 30 | F | - | Occupational therapy |
| D | 30 - 40 | F | - | Arts therapy |
| E | 50 – 60 years | F | 13y | Behavioral sciences, social work, nursing |
| F | 40 - 50 | F | 19y | Speech therapy |
| G | 40 - 50 | F | 23y | Occupational therapy |

Characteristics of expert group: Educational scientists

| **Educational scientist** | **Age (range)** | **Gender** | **Years of experience in education** | **Professional background** |
| --- | --- | --- | --- | --- |
| A | - | - | 35 years | Medicine, medical educational scientist |
| B | 40-50y | M | 14 years | Educational scientist, assessment expert |
| C | 30-40y | F | 14 years | Assessment expert |
| D | 30-40y | M | 7 years | Instructional design expert |
| E | 50-60y | F | 30 years | Assessment expert, educational scientist |
| F | 50-60y | F | 20 years | Health sciences |
| G | 30-40y | F | 9 years | Educational scientist |

Characteristics of expert group: Students

| **Student** | **Age** | **Gender** | **Educational programme** | **Experience in IPE** | **Previous education** |
| --- | --- | --- | --- | --- | --- |
| A | 22 | F | Occupational therapy year 3 | Participation in IP educational modules  Participation in IP minor | - |
| B | 20 | F | Nursing year 3 | Participation in IP educational modules Student tutor in IP educational modules  IP internships  Participation in IP project | - |
| C | 27 | F | Nursing year 2 | Participation in IP educational modules  IP internship | Medical management assistant |
| D | 19 | F | Nursing year 3 | IP educational modules  IP team meetings | - |
| E | 20 | F | Nursing year 3 | IP educational modules  IP internships | - |
| F | 24 | F | Occupational therapy year 3 | IP educational modules  IP minor  IP internships | Physician assistant |
| G | 20 | F | Nursing year 3 | IP educational modules  IP team meetings | - |
| H | 19 | F | Nursing year 3 | IP educational modules  IP internships | - |
| I | 20 | F | Occupational therapy year 3 | IP educational modules  IP minor  IP team meetings | - |
