# Supplementary material for: Design guidelines for assessing students’ interprofessional competencies in healthcare education: a consensus study
Source: Perspect Med Educ. 2022 Oct 12;11(6):316–24. doi: 10.1007/s40037-022-00728-6 (PMC9743853; doi:10.1007/s40037-022-00728-6)
Supplement: Supplementary file 2 — ESM 2 Qualitative interview guide used in this study [file 40037_2022_728_MOESM2_ESM.doc]

**ESM 2**

**Interview guide NGT sessions**

***Assessment tasks***

**Main question** (each person gets the chance to answer one by one, 2 minutes per person)

- Which requirements do you pose for the design of an interprofessional assessment task?

**In depth questions**

- What do interprofessional assessment tasks look like?
- Which features do they have?
- What makes a task an interprofessional task?
- What evidence of interprofessional competence should an interprofessional task yield? Processes and/or products?

**Discussion & clarification**

Are there any other ideas regarding important aspects of interprofessional assessment tasks that you would like to share with us?

***Assessors***

**Main question** (each person gets the chance to answer one by one, 2 minutes per person)

- Which requirements do you pose for the assessors for the interprofessional assessment?

**In depth questions**

- Who are IP assessors and what are their characteristics?
- What should the pool of assessors look like?
- How should a pool of assessors be composed?
- What do assessors need to reach a decision about the IP competence of the students?

**Discussion & clarification**

Are there any other ideas regarding important aspects of interprofessional assessors that you would like to share with us?

**Assessment model**

**Main question** (each person gets the chance to answer one by one, 2 minutes per person)

- Which requirements do you pose for a tool to assess interprofessional competence?

**In depth questions**

- What should be included in the assessment format that assessors use for the assessment of interprofessional competence?
- What makes an assessment format interprofessional?
- What would the tools look like that assessors use in order to reach a decision about the acquisition of IP competence?

**Discussion & clarification**

Are there any other ideas regarding important aspects of interprofessional assessment models that you would like to share with us?
