# Supplementary material for: Design guidelines for assessing students’ interprofessional competencies in healthcare education: a consensus study
Source: Perspect Med Educ. 2022 Oct 12;11(6):316–24. doi: 10.1007/s40037-022-00728-6 (PMC9743853; doi:10.1007/s40037-022-00728-6)
Supplement: Supplementary file 3 — ESM 3 Figure that gives an overview of the categories per assessment element tasks, assessors, and procedures, that followed the analysis of the data [file 40037_2022_728_MOESM3_ESM.docx]

**ESM 3**

Figure Analysis

**Fig. S1**

*Nine categories following the analysis*
